# Evaluation design of the Social Engagement Framework for Addressing the Chronic-disease-challenge (SEFAC): a mindfulness-based intervention to promote the self-management of chronic conditions and a healthy lifestyle

**DOI:** 10.1186/s12889-019-6979-7

**Published:** 2019-05-30

**Authors:** Xuxi Zhang, Siok Swan Tan, Irene Fierloos, Oscar Zanutto, Tamara Alhambra-Borrás, Vanja Vasiljev, Scott Bennett, Tasos Rentoumis, Antonella Buranello, Stefania Macchione, Ellen Rouwet, Amy van Grieken, Hein Raat

**Affiliations:** 1 Department of Public Health, Erasmus University Medical Center, Rotterdam, The Netherlands; 2 European Project Office Department, Istituto per Servizi di Ricovero e Assistenza agli Anziani (Institute for Hospitalization and Care for the Elderly), Treviso, Italy; 3 Polibienestar Research Institute, University of Valencia, Valencia, Spain; 4 Faculty of Medicine, Department of Social Medicine and Epidemiology, University of Rijeka, Rijeka, Croatia; 5 Veor Surgery, Camborne, UK; 6 Alliance for integrated care, Athens, Greece; 7 Department of Surgery, Erasmus University Medical Center, Rotterdam, The Netherlands

**Keywords:** Prevention, Self-management, Type 2 diabetes, Cardiovascular disease, Mindfulness, Lifestyle, Social engagement, ICT support

## Abstract

**Background:**

The Social Engagement Framework for Addressing the Chronic-disease-challenge (SEFAC) project intends to empower citizens at risk of or with type 2 diabetes (T2DM) and/or cardiovascular disease (CVD) to self-manage their chronic conditions through the SEFAC intervention. The intervention combines the concepts of mindfulness, social engagement and information and communication technology support, in order to reduce the burden of citizens with chronic conditions and to increase the sustainability of the health system in four European countries.

**Methods:**

A prospective cohort study with a 6-month pre-post design will be conducted in four European countries: Croatia, Italy, the Netherlands and the United Kingdom. A total of 360 community-dwelling citizens ≥50 years of age will be recruited; 200 citizens at risk of T2DM and/or CVD in the next 10 years (50 participants in each country) and 160 citizens with T2DM and/or CVD (40 participants in each country). Effects of the intervention in terms of self-management, healthy lifestyle behavior, social support, stress, depression, sleep and fatigue, adherence to medications and health-related quality of life will be assessed. In addition, a preliminary cost-effectiveness analysis will be performed from a societal and healthcare perspective.

**Discussion:**

The SEFAC project will further elucidate whether the SEFAC intervention is feasible and (cost-) effective among citizens at risk of and suffering from T2DM and/or CVD in different settings.

**Trial registration:**

ISRCTN registry number is ISRCTN11248135. Date of registration is 30/08/2018 (*retrospectively registered*).

**Electronic supplementary material:**

The online version of this article (10.1186/s12889-019-6979-7) contains supplementary material, which is available to authorized users.

## Background

Persons with a chronic condition are responsible for the management of their chronic condition everyday [1]. Successful self-management of chronic conditions could help citizens handle their life with independence to some extent despite their medical condition and to feel healthy despite their limitations [2]. Moreover, within the context of the overloaded healthcare and welfare systems, the ability of citizens with a chronic condition to take care of themselves for as long as possible has become increasingly important [1, 2].

Several concepts have recently been explored as a basis to define the most effective and efficient model to deal with the chronic condition challenge [3]. One of these concepts concerns mindfulness. A review of 15 studies suggested that mindfulness-based stress reduction interventions could help participants with chronic conditions to better cope with symptoms and better achieve overall well-being, quality of life and health outcomes [4]. Some studies indicate that a mindfulness intervention is an effective tool for diabetes as well as chronic low back pain self-management [5, 6].

A second concept concerns social engagement. Social engagement programmes provide practical support to help citizens achieve aspirations and make them better connected to their community. One example of a social engagement programme is the Newquay Pathfinder Programme [7]. Important conceptual elements of this programme include shaping services around people and communities, motivating people to achieve their aspirations through a ‘guided conversation’ and the use of volunteers [7, 8].

Information and communication technology (ICT) (for instance, a telephone-based interactive system or an application on smartphone) is the third concept which is considered as an important enabler of self-management partnership [1]. This means that people with chronic conditions can self-manage their health using ICT and health professionals are consulted to support them in this role [1, 9, 10]. Previous studies indicate that ICT support improves the self-management of citizens with chronic conditions [11, 12].

Numerous studies have demonstrated the effectiveness of self-management programmes [13–15]. However, most studies have focused on a specific concept and/or a specific chronic condition [16]. Furthermore, cross country comparisons of the effectiveness of these programmes are recommended as well as cost-efficiency data regarding these self-management programme [17].

## The SEFAC project

The Social Engagement Framework for Addressing the Chronic-disease-challenge (SEFAC) project was set up to respond to the call of the Third EU Health Programme (2014–2020; PJ-04-2016: Support to Member States and stakeholders to address the chronic disease challenge; http://sefacproject.eu). The aim of the SEFAC project is to empower citizens ≥50 years of age at risk of or with type 2 diabetes (T2DM) and/or cardiovascular disease (CVD) to self-manage their chronic conditions through the SEFAC intervention which combines the concepts of mindfulness, social engagement as well as ICT support. Furthermore, the project will evaluate (cost) effectiveness, which will provide insight in costs of potential policies contributing to the prevention of chronic conditions. In this project, study sites in four European countries will implement the SEFAC intervention: Rijeka in Croatia, Treviso in Italy, Rotterdam in the Netherlands and Camborne in the United Kingdom.

## Objectives

The main objective of this paper is to evaluate the SEFAC intervention in terms of benefits for the target population (citizens ≥50 years of age at risk of or with T2DM and/or CVD). The following research questions will be answered:

1. What are the effects of the SEFAC intervention for participants in terms of self-management, healthy lifestyle behavior, social support, stress, depression, sleep and fatigue, adherence to medications and health-related quality of life (HRQoL)?

2. What are the societal cost savings of the SEFAC intervention in terms of reducing healthcare utilization and productivity losses among the target population?

3. To what extent is the target population satisfied with the SEFAC intervention as a whole and with its three specific elements (mindfulness, social engagement and ICT support)?

## Study hypotheses

Our hypothesis is that the SEFAC intervention will improve the self-management skills of participants, promote more favorable lifestyle behaviors, improve social support, reduce participants’ stress, depression, sleeping problems and fatigue and improve participants’ adherence to medication and HRQoL at 6 month of follow-up compared to baseline. In addition, we hypothesize that society will benefit from the intervention through to a reduced use of healthcare resources and greater productivity. Finally, we hypothesize to reach a satisfaction score of 7 or higher on a 1–10 scale for the SEFAC intervention as a whole, with higher scores representing greater satisfaction.

## Methods/design

### The SEFAC intervention

**Fig. 1:**
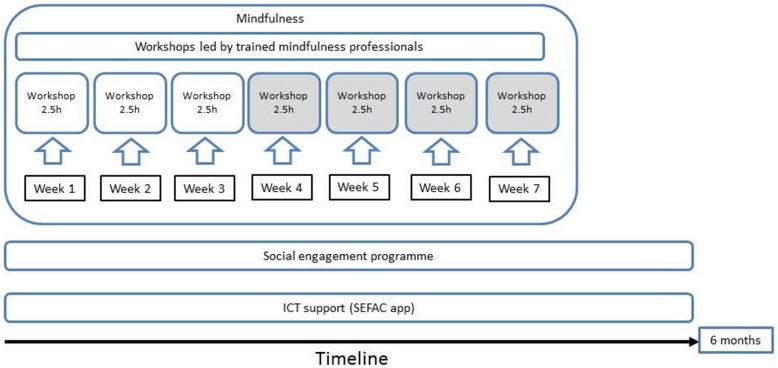
The SEFAC intervention

The SEFAC intervention was designed and developed by partners of the SEFAC project and includes the concepts of mindfulness, social engagement as well as ICT support (Fig. 1), which are offered to participants in parallel.

Mindfulness training is offered in a series of 3 to 7 workshops, 2.5 h each, which will be held once a week for 3 to 7 weeks. Every training will be led by trained mindfulness professionals. The training includes three ‘obligatory’ workshops on training mind and body for health and wellbeing, healthy habits and a healthy mindset as well as four voluntary workshops on healthy eating, healthy physical activity, healthy relationships and healthy life with chronic conditions. The number of participants per training will be no more than 30. Over the workshops, participants will learn to foster greater awareness of present moment experience to help them better manage life’s ups and downs, support a healthy lifestyle and enhance the quality of daily life.

In parallel to the mindfulness training, participants are invited to enroll in the social engagement programme of the SEFAC project which is based on the Newquay Pathfinder Programme [7]. The precise role of the volunteers may differ depending on the geographical, cultural and social context of the four study sites. At least, volunteers help citizens identify ways to build self-confidence and self-reliance through guided conversations [7]. In addition, they may support the mindfulness training and provide practical help in adopting major lifestyle changes and in getting better connected to their community.

Finally, participants will be invited to download the free SEFAC app on their mobile phone and use it as ICT support for 6 months, starting from the first workshop. The SEFAC app is a multi-modular tool that has been developed for the android operating system. The app aims to support change of lifestyle behaviors among people with and without chronic conditions, according to the stage of change the individual is in at a particular point in time. Participants are encouraged to engage in the practices, lessons, tips and reflections offered through the app (see Additional file 1: Figure S1; Additional file 2: Figure S2; Additional file 3: Figure S3; Additional file 4: Figure S4; Additional file 5: Figure S5).

### Study design, setting and procedures

A prospective cohort study with a 6-month pre-post design will be conducted [18]. Six-month follow-up data of participants will be compared with the same participant’s baseline data. The study protocol has been reviewed by the Ethical Review Boards at the study sites in Rijeka, Treviso, and Rotterdam; at the study site in Camborne the decision tool of the NHS Health Research Authority was applied in accordance with the applicable regulations in the UK. See Declaration section. In all cases, written informed consent is obtained before participants enter the study.

In each study site, we will recruit community-dwelling citizens over 50 years old using different strategies taking the capacity, organizational and environmental characteristics of the 4 study sites in consideration, as described below.

***Rijeka*** is a port city in the Republic of Croatia with a population of 128,384 [19].Participants will be recruited from public health events where free health checks are provided, including measurement of blood pressure and blood glucose, as well as through free community exercise programmes. Interested citizens can talk about the risk of developing T2DM and/or CVD with a health professional. Eligible citizens are informed about the SEFAC project and are invited to provide written informed consent and to participate in the study.

***Treviso*** is a city in the Veneto region in northeast Italy with 85,200 inhabitants [20]. Participants will be recruited from open events and through announcements on health-related social network platforms. Interested citizens can talk face-to-face with health professionals about the risk of developing T2DM and/or CVD, and can be suggested to visit their general practitioner (GP). Eligible citizens are informed about the SEFAC project and are invited to provide written informed consent and to participate in the study.

***Rotterdam*** is a port city in the Netherlands with a population of 644,527 [21]. Participants will be recruited from open community events and public announcements. Citizens are informed about the SEFAC project in-person and/or via the SEFAC website. Interested citizens can express their interest to participate online, by e-mail and in a conversation with a health professional, face-to-face or by telephone. Eligible citizens are invited by the research team to provide written informed consent and to participate in the study.

***Camborne*** is a town in South West England with a population of 20,436 [22]. Participants will be recruited by informing and inviting visitors of the Veor Surgery, a general practitioner practice. Recruitment will also take place through open events. Eligible participants will receive information about the SEFAC project and are invited to provide written informed consent and to participate.

### Study population and eligibility to participate in the study

We aim to include 360 participants in total (90 participants in each study site). The target population consists of community-dwelling citizens ≥50 years of age, of which 200 participants at risk of T2DM and/or CVD in the next 10 years (50 participants in each study site) and 160 participants with T2DM and/or CVD (40 participants in each study site). Citizens are not eligible to participate when they are diagnosed with mild or serious cognitive impairment, terminally ill or scheduled to enter secondary or tertiary care settings for a long period of time, lack the basic knowledge of the local language or are not able to make an informed decision regarding participation in the study.

### Data collection

Data will be collected from participants before the start of the first workshop (baseline, T0) and at 6 months (T1) with the use of a questionnaire. The instruments used for the outcome measures are described in measurements section. The instruments or items without validated translations are translated by translators. The study team discussed the translations and adapted the translation when needed.

### Measurements

Our objective is to evaluate the effects of the SEFAC intervention on self-management, healthy lifestyle behavior, social support, stress, depression, sleep and fatigue, adherence to medications and HRQoL. Self-management is measured with General Self-efficacy Scale (GES) [23] as well as the short 6-item version of the Chronic Disease Self-Efficacy instrument (CDSE-6) [24] which measure the confidence in one’s ability to deal with health problems. The CDSE-6 covers domains that are common across many chronic conditions, such as symptom control, role function, emotional functioning and communicating with physicians.

With respect to healthy lifestyle behavior, we will assess physical activity, healthy eating, sedentary behavior, smoking and alcohol use. Physical activity is measured with six items on physical exercise [24] and five items of The Physical Exercise Self-Efficacy Scale (PESES) [25]. Healthy eating is measured with three items on the intake of fruits, vegetables and breakfast and five items of The Nutrition Self-Efficacy Scale (NSES) [26]. Sedentary behavior is measured with one item from the International Physical Activity Questionnaire (IPAQ) [27], current smoking is assessed with a single yes/no question and the frequency of alcohol use is determined with one item from the AUDIT-C [28].

Social support is measured with the 3-item Oslo Social Support scale (OSS-3), regarding the primary support group, interest and concern shown by others and ease of obtaining practical help [29]. Stress is measured with the 10-item Perceived Stress Scale (PSS-10) [30]. Depression is measured with the 8-item Patient Health Questionnaire depression scale (PHQ-8) [31]. Sleep and fatigue are measured with visual analog scales, ranging from 0 (no sleeping problem/fatigue) to 10 (severe sleeping problem/fatigue).

Adherence to medication is measured with six items from the Short Medication Adherence Questionnaire (SMAQ) [32], a short tool based on questions posed directly to the participant regarding his/her medication-taking habits.

HRQoL is measured with the 12-item Short-Form health survey (SF-12) [33] and the EuroQol- 5 Dimensions- 5 level (EQ-5D-5 L) instrument [34]. The SF-12 is a patient-reported survey which includes both a physical dimension (physical functioning, role-physical, pain and general health) and a mental dimension (vitality, social functioning, role-emotional and mental health). SF-12 scores can be summarized in the Physical Component Summary (PCS) and the Mental Component Summary (MCS), ranging from 0 (worst) to 100 (best quality of life) [33]. The EQ-5D-5 L is often used in the Quality-Adjusted Life Year calculation to determine the cost-effectiveness of an intervention. It has five dimensions: mobility, self-care, activity, pain and anxiety. Each dimension has five levels, ranging from no problems (level 1) to serious problems (level 5). Hence, the EQ-5D-5 L has 3125 possible health states. Utility values for these health states are available for the study sites of each participating country [34]. As part of the EQ-5D-5 L, participants are also asked to indicate their experienced current health state on a visual analog scale, 0 being the worst imaginable health and 100 being the best imaginable health.

Additionally, we will evaluate healthcare utilization and productivity losses. Healthcare utilization is measured with four questions from the Self-Management Resource Center (SMRC) Health Care Utilization questionnaire regarding doctor appointments, the use of hospital emergency rooms and hospital admissions [35, 36]. Productivity losses are measured with two domains from the Productivity Costs Questionnaire (PCQ): lost productivity at paid work due to absenteeism (6 items) and lost productivity at unpaid work (3 items) [37].

Socio-demographic characteristics include age, gender, country of birth, marital status, household composition, education level, employment situation and health conditions. There is an open box at the end of the questionnaire for any additional remarks.

The follow-up questionnaire at 6 months (T1) will be identical to the baseline questions except for the addition of questions on the satisfaction of the target population with the intervention. In the T1-questionnaire, we will add 6 items to rate the satisfaction with the whole SEFAC intervention as well as specific concepts (mindfulness, social engagement and ICT support) on a scale from 1 to 10.

### Power considerations

The power considerations are conducted according to the methods of a previous study [38]. We will include net 113 participants at T0 in each study site (4 study sites * 113 = 452 study participants). When the loss to follow-up between T0 and T1 will be 20%, we will have complete data of 360 participants at T1. Assuming equal standard deviations (SD) at T0 and T1, an alpha of 0.05 and power of 0.80, and taking into account the cluster design (4 participating study sites) with an average cluster size of 90 participants (360/4) and an intra-class correlation coefficient of 0.02, a difference of 0.24 SD between T0 and T1 can be established regarding the continuous outcome measures for this expected sample size and under these conditions. For instance, regarding HRQoL as measured by the SF-12, a difference of 2.74 points can be established between T0 and T1 for the PCS (SD = 11.4) and 2.86 points for the MCS (SD = 11.9) [39].

### Statistical analysis

Descriptive statistics will describe characteristics of participants in the total study population and in each study site. In order to evaluate differences between T0 and T1 measurements, multiple linear regression analyses (for continuous outcome variables) and multiple logistic regression analyses (for dichotomous variables) will be adopted in the total study population. In addition, the analyses will be done for each study site separately, and possibly other subgroups analyses will be performed through formal interaction tests for variables that will likely effect the intervention itself, such as age, gender and education level.

A preliminary cost-effectiveness analysis will be performed with the baseline measurement as control group from a societal and healthcare perspective. Healthcare costs for individual participants will be determined by multiplying resource use with corresponding unit prices for 2017, including doctor appointments, hospital emergency rooms and hospital admissions. Productivity losses for individual participants (lost productivity at paid work due to absenteeism and lost productivity at unpaid work) will follow from the PCQ. Utility values will be obtained through the EQ-5D-5 L instrument.

### Dissemination

An Advisory Board with experts from five countries (China, Croatia, Finland, the Netherlands and Sweden) has been set up. The Advisory Board will provide critical suggestions and comments throughout the project. The project team will disseminate the scientific project results through publications in scientific peer-reviewed journals and conferences. We adopt the project website (http://sefacproject.eu/) to further disseminate the key findings of our project to all stakeholders. The European Local Inclusion and Social Action Network (ELISAN) will disseminate the project results through social media.

## Discussion

This paper describes the design of a prospective cohort study which aims to evaluate the effects of the SEFAC intervention for citizens at risk of or with T2DM and/or CVD on self-management, healthy lifestyle behaviors, social support, stress, depression, sleep and fatigue, adherence to medications and HRQoL as well as the (cost-) effectiveness of the SEFAC intervention.

Strengths of the study are that, to our knowledge, this study is the first to develop and implement an intervention combining the concepts of mindfulness, social engagement and ICT support in Europe. Our study may provide evidences on the generalizability of the intervention in different European countries through recruiting the target population in different settings. Additionally, the SEFAC project will provide information on cost-effectiveness of self-management programmes to fulfill the gap of limited data in this area.

The study also has some limitations and challenges. Firstly, recruiting citizens at risk of or with T2DM and/or CVD may be a challenge. In order to increase the participation rates, open events aim at recruiting participants will be held according to the capacity, organizational and environmental characteristics of the 4 study sites. Secondly, it was not practicable to include a control group. To ensure that a citizen would not feel excluded, we prefer to offer the intervention to all citizens that meet our criteria. Instead, we apply a 6-month pre-post design, using the baseline measurement as the ‘control group’. Thirdly, we will try to capture the most important confounding factors in our questionnaire. However, it is still possible that we miss relevant variables.

Chronic conditions are the main cause of morbidity and mortality in Europe and due to their social impact and economic implications, their prevention and management are important challenges in realizing the sustainability of health systems in Europe. By combining mindfulness training, social engagement and ICT support, we expect the SEFAC intervention to be a feasible and cost-effective programme to promote self-management and self-care of citizens at risk of and suffering from chronic conditions.

## Additional files

Additional file 1: **Figure S1.** Screen capture of SEFAC app: part 1 (DOCX 76 kb)

Additional file 2: **Figure S2.** Screen capture of SEFAC app: part 2 (DOCX 66 kb)

Additional file 3: **Figure S3.** Screen capture of SEFAC app: part 3 (DOCX 117 kb)

Additional file 4: **Figure S4.** Screen capture of SEFAC app: part 4 (DOCX 91 kb)

Additional file 5: **Figure S5.** Screen capture of SEFAC app: part 5 (DOCX 68 kb)

## Abbreviations

CDSE-6: Short 6-item version of the Chronic Disease Self-Efficacy instrument
CVD: Cardiovascular Disease
ELISAN: European Local Inclusion and Social Action Network
EMC: Erasmus MC University Medical Center
EQ-5D-5 L: EuroQol- 5 Dimensions- 5 level
EU: European Union
GSE: General Self-Efficacy scale
HRQoL: Health Related Quality of Life
ICT: Information and Communication Technology
IPAQ: International Physical Activity Questionnaire
NSES: Nutrition Self-Efficacy Scale
OSS-3: Oslo Social Support scale
PCQ: Productivity Costs Questionnaire
PESES: Physical Exercise Self-Efficacy Scale
PHQ-8: 8-item Patient Health Questionnaire depression scale
PSS-10: 10-item Perceived Stress Scale
SEFAC: Social Engagement Framework for Addressing the Chronic-disease-challenge
SF-12: 12-item Short Form health survey
SMAQ: Short Medication Adherence Questionnaire
SMRC: Self-Management Resource Center
T2DM: Type 2 Diabetes Mellitus
