# Supplementary figures and images for: Evaluation design of the Social Engagement Framework for Addressing the Chronic-disease-challenge (SEFAC): a mindfulness-based intervention to promote the self-management of chronic conditions and a healthy lifestyle
Source: BMC Public Health. 2019 May 30;19:664. doi: 10.1186/s12889-019-6979-7 (PMC6543603; doi:10.1186/s12889-019-6979-7)

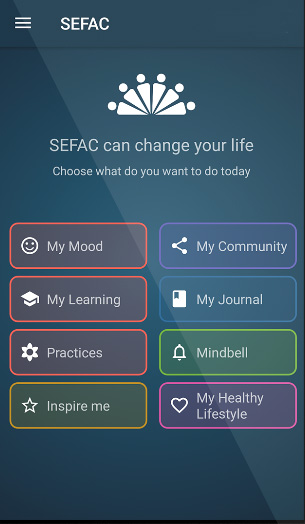


Figure S1 Screen capture of SEFAC app: part 1

Supplement: Supplementary file 1 — Figure S1. Screen capture of SEFAC app: part 1 (DOCX 76 kb) [file 12889_2019_6979_MOESM1_ESM.docx]

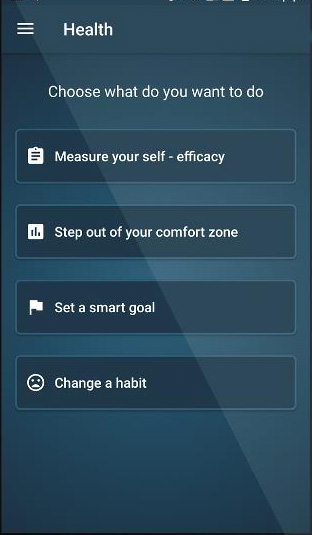


Figure S2 Screen capture of SEFAC app: part 2

Supplement: Supplementary file 2 — Figure S2. Screen capture of SEFAC app: part 2 (DOCX 66 kb) [file 12889_2019_6979_MOESM2_ESM.docx]

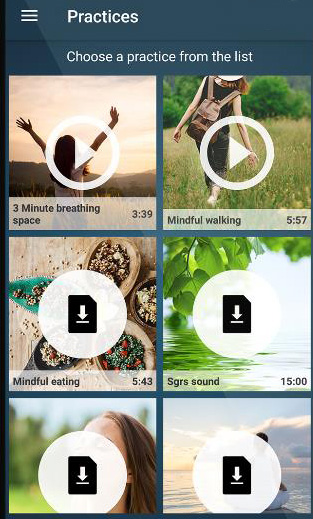


Figure S3 Screen capture of SEFAC app: part 3

Supplement: Supplementary file 3 — Figure S3. Screen capture of SEFAC app: part 3 (DOCX 117 kb) [file 12889_2019_6979_MOESM3_ESM.docx]

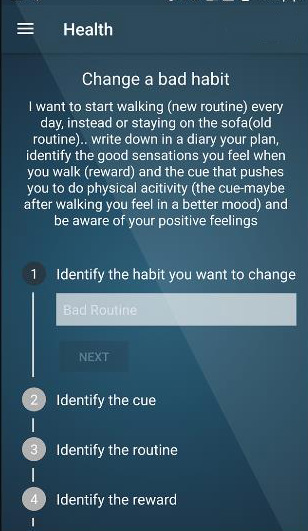


Figure S4 Screen capture of SEFAC app: part 4

Supplement: Supplementary file 4 — Figure S4. Screen capture of SEFAC app: part 4 (DOCX 91 kb) [file 12889_2019_6979_MOESM4_ESM.docx]

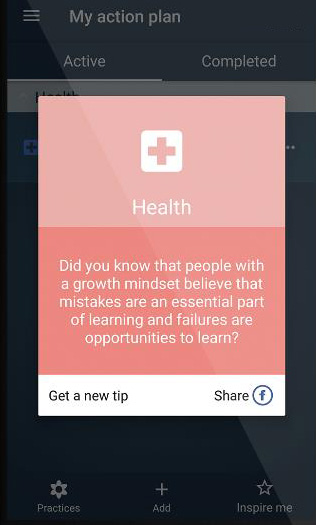


Figure S5 Screen capture of SEFAC app: part 5

Supplement: Supplementary file 5 — Figure S5. Screen capture of SEFAC app: part 5 (DOCX 68 kb) [file 12889_2019_6979_MOESM5_ESM.docx]
